# Evaluation Framework for Successful Artificial Intelligence–Enabled Clinical Decision Support Systems: Mixed Methods Study

**DOI:** 10.2196/25929

**Published:** 2021-06-02

**Authors:** Mengting Ji, Georgi Z Genchev, Hengye Huang, Ting Xu, Hui Lu, Guangjun Yu

**Affiliations:** 1 School of Public Health Shanghai Jiao Tong University School of Medicine Shanghai China; 2 Ren Ji Hospital School of Medicine Shanghai Jiao Tong University Shanghai China; 3 Center for Biomedical Informatics Shanghai Children's Hospital Shanghai China; 4 SJTU-Yale Joint Center for Biostatistics Shanghai Jiao Tong University Shanghai China; 5 Bulgarian Institute for Genomics and Precision Medicine Sofia Bulgaria; 6 Department of Bioinformatics and Biostatistics Shanghai Jiao Tong University Shanghai China; 7 Shanghai Children’s Hospital Shanghai Jiao Tong University Shanghai China

**Keywords:** artificial intelligence, AI, clinical decision support systems, evaluation framework

## Abstract

**Background:**

Clinical decision support systems are designed to utilize medical data, knowledge, and analysis engines and to generate patient-specific assessments or recommendations to health professionals in order to assist decision making. Artificial intelligence–enabled clinical decision support systems aid the decision-making process through an intelligent component. Well-defined evaluation methods are essential to ensure the seamless integration and contribution of these systems to clinical practice.

**Objective:**

The purpose of this study was to develop and validate a measurement instrument and test the interrelationships of evaluation variables for an artificial intelligence–enabled clinical decision support system evaluation framework.

**Methods:**

An artificial intelligence–enabled clinical decision support system evaluation framework consisting of 6 variables was developed. A Delphi process was conducted to develop the measurement instrument items. Cognitive interviews and pretesting were performed to refine the questions. Web-based survey response data were analyzed to remove irrelevant questions from the measurement instrument, to test dimensional structure, and to assess reliability and validity. The interrelationships of relevant variables were tested and verified using path analysis, and a 28-item measurement instrument was developed. Measurement instrument survey responses were collected from 156 respondents.

**Results:**

The Cronbach α of the measurement instrument was 0.963, and its content validity was 0.943. Values of average variance extracted ranged from 0.582 to 0.756, and values of the heterotrait-monotrait ratio ranged from 0.376 to 0.896. The final model had a good fit (*χ_26_^2^*=36.984; *P*=.08; comparative fit index 0.991; goodness-of-fit index 0.957; root mean square error of approximation 0.052; standardized root mean square residual 0.028). Variables in the final model accounted for 89% of the variance in the user acceptance dimension.

**Conclusions:**

User acceptance is the central dimension of artificial intelligence–enabled clinical decision support system success. Acceptance was directly influenced by perceived ease of use, information quality, service quality, and perceived benefit. Acceptance was also indirectly influenced by system quality and information quality through perceived ease of use. User acceptance and perceived benefit were interrelated.

## Introduction

### Clinical Decision Support Systems

Clinical decision support systems are computer-based enterprise systems designed to utilize massive data, medical knowledge, and analysis engines as well as to generate patient-specific assessments or recommendations to health professionals in order to assist clinical decision making through human–computer interaction [1,2]. These systems provide services ranging from simple reminders to complex risk prediction [3] and support health care providers in diagnosis, treatment decisions, and population health management. Clinical decision support systems assist one or more levels of decision making: alerting, interpreting, critiquing, assisting, diagnosing, and managing [4]. Diagnostic support systems are a subset of clinical decision support systems that are specifically designed to support clinician in diagnosing patients [5]. Artificial intelligence (AI)–enabled clinical decision support systems combine the knowledge reasoning techniques of AI and the functional models of clinical decision support systems [6].

### AI-Enabled Clinical Decision Support Systems: Characteristics, Usage, and Benefits

AI-enabled clinical decision support systems include an intelligent component [6], and in comparison to traditional clinical decision support systems, represent a paradigm shift. They are designed to aid clinicians by converting raw medical-related data, documents, and expert practice into a set of sophisticated algorithms, applying techniques such as machine learning, knowledge graphs, natural language processing, and computer vision so that users find suitable solutions to their medical problems and make clinical decisions [7]. AI-enabled clinical decision support systems have the potential to improve clinicians’ performance, quality of health care, and patient safety [8].

Diagnostics are a primary use case of AI-enabled clinical decision support systems, and these systems have been applied in the field of rare disease diagnosis [9], sepsis detection or prediction [10], fracture detection [11], and cancer detection or diagnosis [12,13]. In addition, current AI-enabled clinical decision support systems are also used in medication therapy [14,15] and health care management [16,17].

The greatest benefits of AI-enabled clinical decision support systems reside in their ability to learn from real-world use and experience (ie, training) and their capabilities for improving their performance (ie, adaptation) [18]. By using techniques such as knowledge graphs and natural language processing, AI can deal with large amounts of text classification, information retrieval, and information extraction from the corpora that is provided by hospital electronic health records. Based on structured data, AI can support more comprehensive and more personalized decision-making suggestions for clinicians through techniques such as machine learning. Another benefit is that the functionality and utility from combining clinical decision support systems with AI techniques surpass those of traditional clinical decision support systems, and the system improves and supports the decision-making process by providing intelligent behavioral patterns, with the ability to learn new clinical knowledge [7].

### Need for AI-Enabled Clinical Decision Support System Evaluation

A comprehensive evaluation framework with common elements and interoperability is necessary to serve as a reference for AI-enabled clinical decision support system design and evaluation, with focuses on cross-disciplinary communication and collaboration, and there is a pressing need to develop robust methodologies and empirically based tools for such evaluation. The factors driving this need are the uncertain added value of AI-enabled clinical decision support system implementation, lack of attention, and the possible benefits of comprehensive evaluation implementations.

First, the added value of AI-enabled clinical decision support system implementations in a clinical setting is not firmly established, though evidence exists that such implementations offer potential benefit to patients, clinicians, and health care in general [19]. Introducing this type of system in clinical settings is not without risk [8]. Similar to any other newly introduced technology, AI-enabled clinical decision support systems may disrupt clinical service, threaten patient safety [20], and cause more negative than positive impacts [19]. As a result, there are concerns that AI-enabled clinical decision support system implementation can introduce new errors and have unintended consequences [21]. Additionally, the effect of these systems on clinical, social, and economic outcomes is still controversial which highlights the need to evaluate recognized value parameters [22]. Second, attention to evaluation of clinical decision support systems, in general, and AI-enabled clinical decision support systems, in particular, remains weak [23], which has resulted in a paucity of data on safety, effectiveness, cost benefits, and impacts of AI-enabled clinical decision support systems on patients and health systems [24,25]. Finally, the evaluation of AI-enabled clinical decision support systems is a learning and knowledge-gaining process, and it also helps to identify the gaps to be filled [26]. Findings of comprehensive evaluations could be used to help improve implementations [27].

### AI-Enabled Clinical Decision Support System Evaluation Methodologies

The approach to AI-enabled clinical decision support system evaluation is influenced by a sociotechnical regime, which informs and guides the development of the robust and focused evaluation method of this study. It has increasingly been acknowledged that evaluations of such systems are based on a sociological understanding of the complex practices in which the information technologies are to function [28]. A careful balance between social and technical value is required in order to ensure that unwanted consequences do not pose a threat to patients [26] and clinical practices.

A well-defined success measure, based on users’ perspectives, that specifies aspects of AI-enabled clinical decision support systems that determine their success [29] is critical for a robust performance and usefulness evaluation framework. Due to the user-centric nature of information system development and evaluation [30,31], evaluation of AI-enabled clinical decision support system success aims to recognize factors relevant to user acceptance and utility, thus analysis of articulated users’ opinions is necessary [32]. Clinicians are the direct users of AI-enabled clinical decision support systems; the adoption of the product depends on the individual physicians who decide to use it [5]. In many scenarios, clinicians make decisions for patients, and clinicians are responsible for the medical decisions they make. Predicting and managing users’ attitudes toward AI-enabled clinical decision support systems lead to an in-depth understanding of these systems via situated practice [33] and help developers and medical managers maximize user acceptance. Lack of a well-defined success measure is likely to lead to inappropriate evaluation that does not reflect the clinical impact of AI-enabled clinical decision support systems and may hamper technology advancement[19].

A comprehensive evaluation methodology involves a multidisciplinary process and diverse stakeholder involvement, which, when applied to AI-enabled clinical decision support system evaluation, refers to a mixed methodology not only based on tenets in medicine and information technology but also social and cognitive psychology [30]. Using both qualitative and quantitative methods within a single research project has been shown to provide a richer understanding of a given topic than using solely either a qualitative or quantitative approach, facilitate better and more accurate inferences, and provide an integrated perspective [34]. A similar benefit would likely apply when employing mixed methods in designing an AI-enabled clinical decision support system evaluation scheme.

AI-enabled clinical decision support system interface with a diverse set of clinical and nonclinical users and stakeholders whose inputs are integral to the evaluation process. Health care enterprises are multiprofessional organizations that often include dual hierarchical structures involving clinical practitioners and managers [35], and in such settings, AI-enabled clinical decision support systems are not only tools for clinical practitioners who interact directly with the system (eg, physicians, nurses, pharmacists) but also for nonclinical workers (eg, medical administrators). Additionally, there is still an important group of invisible stakeholders, namely patients, who can be affected by these systems use even without direct interaction. The relationships of such diverse groups of stakeholders can prove to be complex, with competing interests and values; therefore, the views, beliefs, and assumptions of stakeholders must be exposed and considered within the AI-enabled clinical decision support system evaluation process [33,36].

### Objective

We aimed to address the gap in evaluation knowledge and methodologies by identifying which variables influence AI-enabled clinical decision support system success and using these variables to develop a parsimonious evaluation framework. Specifically, we (1) proposed an evaluation framework with 6 variables and hypotheses about interrelationships between the 6 variables based on the literature review, (2) developed and validated an instrument using the 6 variables for assessing the success of diagnostic AI-enabled clinical decision support systems, and (3) tested the hypotheses using path analysis with latent variables in a structural equation model.

## Methods

### Ethics Approval

This study was approved by the Ethics Review Committee, Children’s Hospital of Shanghai/Shanghai Children’s Hospital, Shanghai Jiao Tong University (file number 2020R050-E01).

### Overview

Our study combined qualitative and quantitative methodologies to validate a proposed evaluation framework, which consisted of a model with hypotheses and containing 6 variables.. A Chinese-language measurement instrument was developed with the goal to measure and quantify the 6 variables, following established instrument development paradigm. A literature review and a Delphi process were conducted to develop the measurement instrument items, cognitive interviews, pretest, and web-based survey. Exploratory factor analysis was used to construct the constituent questions of the measurement instrument, reliability and validity tests were performed, and the interrelations of the variables were tested and verified.

### Theory

Evaluation methodologies are informed by a rich corpus of theory, which provides a robust foundation for designing an AI-enabled clinical decision support system evaluation framework. In this study and in previous review work [37], three classic theories were used, namely, the DeLone and McLean Model of Information Systems Success [38], the Information Systems Continuance Model [39,40], and the Information Value Chain Theory [29].

An updated model of information systems success that captures multidimensionality and interdependency was proposed by DeLone and McLean in 2003 [38]; the model is a basic and flexible framework of information system evaluation that can adapt to the complexity of the clinical environment [41-44]. In considering the importance of user acceptance and retention to an information system’s success, the information systems continuance model describes the path from expectation confirmation to the formation of users’ intention to continuance [39]. The information value chain theory underlines decision improvement as the main purpose of technology and provides a mechanism to separate process outcomes from clinical outcomes [45].

### Evaluation Framework Model Variable and Measurement Instrument Item Selection

#### Literature Search

A set of evaluation model variables and a candidate set of medical AI and clinical decision support system evaluation items were collected through a literature review [35]. A broad search strategy was employed, using multiple databases including Cochrane, MEDLINE, EMBASE, Web of Science, PubMed, CINAHL, PsycINFO, and INSPEC. Studies published from January 2009 to May 2020 were utilized to inform the clinical decision support system evaluation items selection and studies published January 2009 to April 2020 for the AI evaluation items discovery. A candidate set of 6 model variables (Multimedia Appendix 1) and a candidate set of 45 evaluation items were identified.

#### Delphi Process

The candidate set of evaluation items was examined and finalized using a Delphi process. Delphi is a structured group communication process, designed to obtain a consensus of opinion from a group of experts [46].

Snowball sampling was used to identify a group of experts. Expert selection criteria were (1) clinical practitioners who worked in a medical specialty at least 10 years, preferably had a PhD (minimum postgraduate qualification), had a professional title at the advanced level or above, had an appointment or affiliation with a professional organization, and had more than 1 year of practical experience (with respect to AI-enabled clinical decision support systems); (2) hospital chief information officers who worked in an information system specialty at least 10 years, had a postgraduate qualification, had a midlevel professional title or above, and had an appointment or affiliation with a professional information system organization; or (3) information technology engineers working in medical information system enterprises who worked in AI or clinical decision support systems at least 5 years, had a postgraduate qualification, and had a midlevel position title or above.

In addition to these selection criteria, a measure of degree of expert authority was introduced to add or remove experts from each round of the Delphi process. The degree of expert authority *C*_r_ was defined *C*_r_ = (*C*_a_ + *C*_s_) / 2, using 2 self-evaluated scores—*C*_a_ is their familiarity with the problem, and *C*_s_ is their knowledge base to judge the program. C_s_ and *C*_a_ ranged between 1 and 5, with a higher value indicating more reliable judgment and more familiarity with the problem. If the self-rated degree of expert authority was >3, the expert was retained, otherwise the expert was removed from group. As a result, a total of 11 experts were selected from diverse areas of expertise and professional focus: clinical practitioners, hospital chief information officers, and information technology engineers working in medical information system enterprises.

The experts were invited to participate in the modified Delphi process via email. Those who accepted were sent an email with a link to the round 1 consultation. Experts were required to provide a relevance score for each item in the candidate set using a 4-point Likert scale (1=not relevant, 2=relevant but requires major revision, 3=relevant but requires minor revision, 4=very relevant and requires no revision). Experts were given 2 weeks to complete each round. A reminder was sent 2 days before the deadline to those who had not completed the survey. The 2-round Delphi process was carried out from May to July 2020.

The content validity was assessed in the last round of the Delphi process. Item-content validity was calculated as the percentage of expert ratings ≥3; if item-content validity was ≥0.8 (ie, expert endorsement), the item was retained. The mean item-content validity, representing the content validity of the measurement instrument of all retained items from the last round was computed. At the end of this step, the set of evaluation items for the measurement instrument were finalized. The final set consisted of 29 evaluation items.

#### Measurement Instrument Refinement

The measurement instrument consisted of the set of evaluation items measured by a web-based survey. A draft set of survey questions was refined by employing cognitive interviews and a pretest. Interviewees (n=5) who were postgraduates majoring in health informatics or end-users of AI-enabled clinical decision support systems (ie, clinicians) were asked to verbalize the mental process entailed in providing answers. The pretest included 20 end-users. The interviews and pretest were conducted in July 2020 and aimed to assess the extent to which the survey questions reflected the domain of interest and that answers produced valid measurements. Responses used a Likert scale from 1 (strongly disagree) to 7 (strongly agree). The wording of the questions was subsequently modified based on the feedback from the respondents. The web-based survey was initiated in July and was closed in September 2020.

### Study Population

The evaluation entities chosen in this study were AI-enabled clinical decision support systems designed to support the risk assessment of venous thromboembolism among inpatients: AI-enabled clinical decision support systems that automatically capture electronic medical records based on natural language processing supporting assessment based on individual risk of thrombosis (eg, Caprini scale or Wells scoring), with monitoring of users and reminders sent to users to provide additional data were targeted.

### Survey Participants and Sample Size

Users of target AI-enabled clinical decision support systems who had at least 1 month of user experience were included. The convenience sample participants were based in 3 hospitals in Shanghai that implemented venous thromboembolism risk assessment AI-enabled clinical decision support systems in clinical settings. We appointed an investigator at each hospital site who was responsible for stating the objective of the study, for identifying target respondents, and for monitoring the length of time it took the participants to complete the survey. This was a voluntary survey. The investigators transmitted the electronic questionnaire link to the respondents through the WeChat communication app.

To ensure usability for exploratory factor analysis [47] and to obtain parameter estimates with standard errors small enough to be of practical use in structural equation modeling [48,49], the required sample size was calculated using to participant-to-item ratio (ranging from 5:1 to 10:1), yielding n=150. A response rate ≥70% was targeted to support external validity [50].

### Quality Control Measures

Quality control measures were implemented to ensure logical consistency, with completeness checks before the questionnaire was submitted by the responders. Before submitting, respondents could review or change their answers. In order to avoid duplicates caused by repeat submissions, respondents accessed the survey via a WeChat account. Submitted questionnaires meeting the following criteria were deleted: (1) filling time <100 seconds, or (2) the answer of following 2 questions were contradictory: “How often do you use the AI-enabled clinical decision support systems?” versus “You use the AI-enabled clinical decision support systems frequently.” Finally, we asked the point-of-contact individuals in each hospital to send online notifications to survey respondents at least 3 times at regular intervals in order to improve the response rate.

### Statistical Analysis

#### Overview

Statistical analyses were performed (SPSS Amos, version 21, IBM Corp) to (1) identify items of measurement instrument that were not related to AI-enabled clinical decision support system success for deletion, (2) explore the latent constructs of the measurement instrument, and (3) evaluate reliability and validity of the measurement instrument.

#### Measurement Instrument Item Reduction

Critical ratio and significance were calculated using independent *t* tests between high- (upper 27%) and low- (lower 27%) score groups. Item-scale correlation was calculated using Pearson correlation. Corrected item-to-total correlations and the effect on Cronbach α if an item was deleted were calculated using reliability analysis. Item-scale correlation and corrected item-to-total correlations were indications of the degree to which each item was correlated with the total score. Criteria for potential elimination were (1) nonsignificant critical ratio (*P*>.05), (2) item-scale correlation <0.40, (3) corrected item-to-total correlation <0.40, (4) an increased α if the item was deleted [51,52], that is, if α increased with an item removed, we considered removal of the item from the measurement instrument [49].

#### Latent Construct of Measurement Instrument

Construct of the measurement tool was tested using exploratory factor analysis. Principal component analysis was applied for factor extraction, and the Promax with Kaiser normalization rotation strategy was used to redefine the factors to improve their interpretability. The cutoff strategy was based on verify if the data set was suitable for exploratory factor analysis—the Bartlett test of sphericity should be statistically significant (*P*<.05) and a Kaiser-Meyer-Olkin value ≥.60 is considered mediocre [49], a value ≥.90 is marvelous [53]. Only factors with an eigenvalue ≥0.50 were retained.

#### Reliability and Validity of Measurement Instrument

Cronbach α coefficients were calculated to assess internal consistencies of the scale and each subscale; values >.80 are preferred [49,50]. Convergent validity and discriminant validity were tested using maximum likelihood estimation confirmatory factor analysis in structural equation modeling. Average variance extracted was used as an indicator of convergent validity, and values >.50 were considered acceptable. The heterotrait-monotrait ratio of correlations was used to test discriminant validity. A heterotrait-monotrait ratio value <0.90 provided sufficient evidence of the discriminant validity of constructs [54].

#### Path Analysis

**Figure 1:**
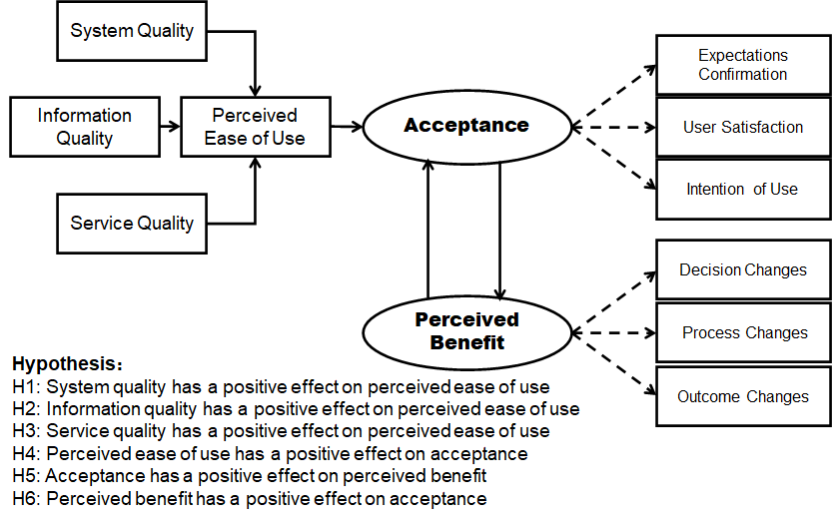
Evaluation model hypotheses.

Interrelationships between variables selected for the evaluation framework were hypothesized in a model (Figure 1). The model was tested using path analysis with latent variables in structural equation modeling. We used the following indicators to assess competence of the model fit: chi-square (significant if *P*>.05), ratio of chi-square to degrees of freedom <2.00), comparative fit index >0.95, goodness-of-fit index >0.95, root mean square error of approximation <0.06, and standardized root mean square residual ≤0.08 [52,55].

## Results

### Measurement Instrument

#### Delphi Process and Evaluation Item Selection

Of the 11 experts invited to participate (Multimedia Appendix 2), all accepted in round 1 (100% response rate) and 10 accepted in round 2 (91% response rate). Most respondents in round 2 (9/10, 90%) identified themselves as expert or very expert (*C*_r_≥4.0) with respect to AI-enabled clinical decision support systems. Consensus was reached in round 2: 29 items obtained at least 80% endorsement (Table 1).

**Table 1:** Accepted items in the Delphi process.

Variables and items			Item-content validity		Critical ratio^a^ (*t* value)		Item-scale correlation^a^		Corrected item-to-total correlation		Cronbach *α* if item was deleted
**Perceived ease of use**											
	Learnability	1.00		6.419		0.643		0.615		.961	
	Operability	1.00		7.384		0.628		0.596		.961	
	User interface	0.90		10.496		0.700		0.669		.960	
	Data entry	1.00		10.530		0.655		0.622		.961	
	Advice display	1.00		7.938		0.655		0.621		.961	
	Legibility	1.00		7.836		0.666		0.641		.961	
**System quality**											
	Response time	1.00		7.826		0.606		0.565		.961	
	Stability	1.00		7.949		0.541		0.498		.962	
**Information quality**											
	Security	1.00		9.247		0.588		0.560		.961	
	Diagnostic performance	1.00		11.346		0.746		0.726		.960	
**Decision changes**											
	Changes in order behavior	0.90		8.593		0.667		0.637		.961	
	Changes in diagnosis	0.90		8.843		0.634		0.600		.961	
**Process changes**											
	Productivity	1.00		11.112		0.726		0.699		.960	
	Effectiveness	1.00		14.078		0.840		0.823		.959	
	Overall usefulness	1.00		13.720		0.826		0.809		.959	
	Adherence to standards	1.00		8.843		0.711		0.688		.960	
	Medical quality	1.00		8.945		0.717		0.696		.960	
	User knowledge and skills	0.80		8.366		0.715		0.692		.960	
**Outcome changes**											
	Change in clinical outcomes	0.90		10.974		0.741		0.719		.960	
	Change in patient-reported outcomes	0.80		10.769		0.716		0.692		.960	
**Service quality**											
	Operation and maintenance	0.90		9.624		0.590		0.555		.961	
	Information updating to keep timeliness	1.00		9.601		0.640		0.614		.961	
**Acceptance**											
	Usage	0.80		4.686		0.323^b^		0.282^b^		.963^b^	
	Expectations confirmation	1.00		14.174		0.856		0.841		.959	
	Satisfaction of system quality	0.80		12.248		0.816		0.798		.959	
	Satisfaction of information quality	0.80		13.437		0.828		0.813		.959	
	Satisfaction of service quality	0.80		11.031		0.737		0.714		.960	
	Overall satisfaction	1.00		15.053		0.873		0.860		.959	
	Intention of use	0.90		13.500		0.855		0.840		.959	

^a^For all values in this column, *P*<.001.

^b^Based on this value, the item meets the standard for potential deletion.

#### Measurement Instrument Formatting

Based on the feedback from the cognitive interviews and pretesting, we made modifications to the wording of 4 items and added explanations to 2 items in order to make them easier to understand. This self-administered measurement instrument with 29 items was used to collected survey data.

### Results of Survey

#### Characteristics of Survey Respondents

Survey responses were collected from a total of 201 respondents (Multimedia Appendix 3) from 3 hospitals in Shanghai, China, of which 156 responses (77.6%) were valid. No data were missing. The ratio of participants to items was 5.4 to 1.

#### Reduction of Items for the Measurement Instrument

One item—usage behavior—was deleted based on item-scale correlation, corrected item-to-total correlation, and effect on Cronbach-α-if-the-item-was-deleted criteria (Table 1).

#### Latent Construct of the Measurement Instrument

Exploratory factor analysis was deemed to be appropriate (Kaiser-Meyer-Olkin .923; *χ_378_^2^*=3859.495; and significant Bartlett test of sphericity, *P*<.001). Eight components, which explained 80.6% of the variance, were extracted (Table 2; Multimedia Appendix 4; Multimedia Appendix 5). For interpretability, we classified decision change, process change and outcome change as one factor—*Perceived benefit*—thereby, the constructs of measurement instrument reflected the 6 variables in the hypothesis model.

**Table 2:** Principal component analysis results.

Component	Extraction			Rotation
	Sums of squared loadings	Variance (%)	Cumulative variance (%)	Sums of squared loadings
Perceived ease of use	14.447	51.596	51.596	11.354
System quality	2.504	8.941	60.537	9.824
Information quality	1.423	5.082	65.620	11.299
Service quality	1.212	4.328	69.948	5.687
Decision change	0.841	3.005	72.953	6.449
Process change	0.779	2.780	75.733	7.736
Outcome change	0.715	2.555	78.288	6.588
Acceptance	0.658	2.350	80.638	5.997

#### Reliability and Validity of Measurement Instrument

The 28-item scale appeared to be internally consistent (Cronbach α=.963). The Cronbach α for the 6 subscales ranged from .760 to .949. Content validity of the overall scale was 0.943. Values of average variance extracted ranged from .582 to .756 and met the >.50 restrictive criterion, which indicated acceptable convergent validity. The values of heterotrait-monotrait ratio ranged from 0.376 to 0.896 and met the <0.90 restrictive criterion, which indicated acceptable discriminant validity of constructs (Table 3, Multimedia Appendix 6).

**Table 3:** Internal consistency, convergent validity, and discriminant validity of constructs.

Variables	Heterotrait-monotrait ratio							Average variance extracted		Composite reliability
	Perceived ease of use	System quality	Information quality	Service quality	Perceived benefit	Acceptance				
Perceived ease of use	1	0.753	0.765	0.412	0.657	0.736	.582		.892	
System quality	0.753	1	0.637	0.376	0.455	0.636	.674		.803	
Information quality	0.765	0.637	1	0.721	0.729	0.767	.620		.760	
Service quality	0.412	0.376	0.721	1	0.654	0.673	.752		.858	
Perceived benefit	0.657	0.455	0.729	0.654	1	0.896	.595		.935	
Acceptance	0.736	0.636	0.767	0.673	0.896	1	.756		.949	

### Model Validation

#### Hypothesized Model Modification

The chi-square of the hypothesized model was significant (*χ_30_^2^*=126.962, *P*<.001; ratio of chi-square over degrees of freedom 4.232). Model fit indices (comparative fit index 0.921; goodness-of-fit index 0.874; root mean square error of approximation 0.144; standardized root mean square residual 0.131) suggested the hypothesized model needed to be modified in order to have a better fitting model: 2 paths, predicting *Acceptance* from *Information quality* and *Service quality*, were added, and one path, predicting *Perceived ease of use* from *Service quality*, was moved, which significantly improved the model and lowered the chi-square values. This meant that in addition to the relationship between *Perceived ease of use* and *Information quality* or *Acceptance*, there was also a direct relationship between *Information quality* and *Acceptance*.

#### Revised Model Fit and Pathway Coefficients

**Figure 2:**
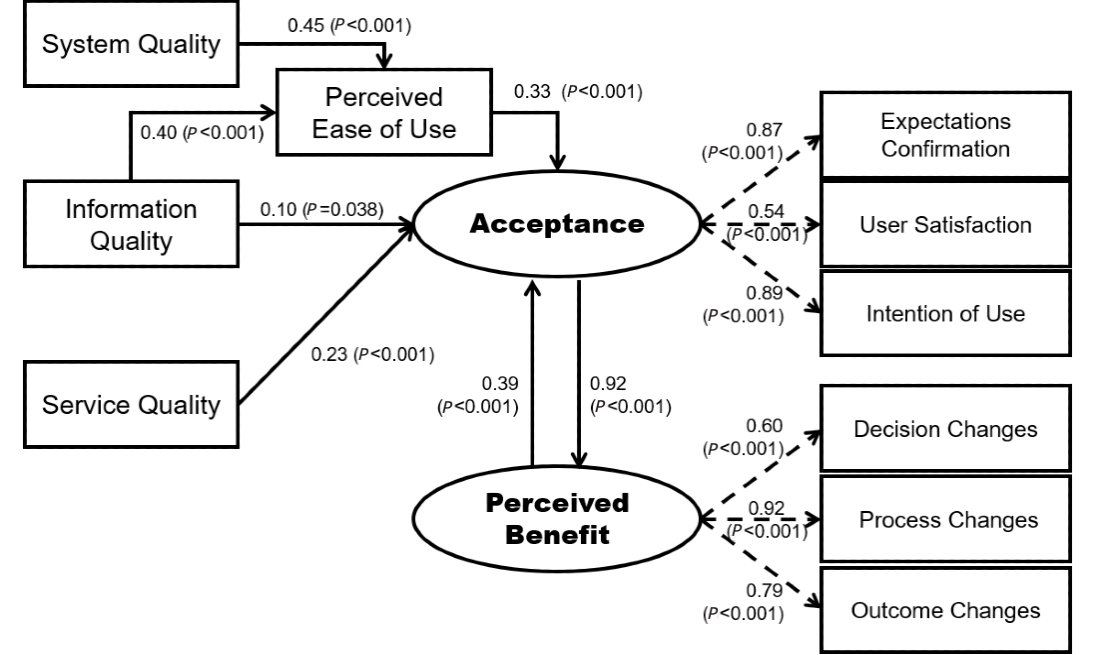
Final evaluation model (comparative fit index 0.991; goodness-of-fit index 0.957; root mean square error of approximation 0.052; standardized root mean square residual 0.028).

The chi-square of the revised model was not significant (*χ_26_^2^*=36.984, *P*=.08; ratio of chi-square over degrees of freedom 1.422). Model fit indices (comparative fit index 0.991; goodness-of-fit index 0.957; root mean square error of approximation 0.052; standardized root mean square residual 0.028) indicated a good-fitting model (Figure 2). All of the path coefficients between measured variables and factors in the final model were significant (2-tailed, *P*<.05). Better *System quality* (*P*<.001) and better *Information quality* (*P*<.001) significantly increased *Perceived ease of use*. Better *Information quality* (*P*=.04), better *Service quality* (*P*<.001), and *Perceived ease of us*e (*P*<.001) significantly increased *Acceptance*. *Acceptance* and *Perceived benefit* were interrelated (Figure 2, Table 4). Variables in the final model accounted for 89% of the variance in *Acceptance* (Table 5). Parameter estimation of error in measurement, standardized total effects, direct effects, and indirect effects are shown in Multimedia Appendix 7-10.

**Table 4:** Parameter estimation for path coefficients.

Pathway		Regression weights	Standardized regression weights	Standard error	Critical ratio	*P* value
Perceived ease of use	System quality	0.292	0.446	0.041	7.139	<.001
Perceived ease of use	Information quality	0.378	0.405	0.058	6.484	<.001
Acceptance	Information quality	0.117	0.099	0.057	2.070	.04
Acceptance	Service quality	0.235	0.232	0.052	4.525	<.001
Acceptance	Perceived ease of use	0.413	0.325	0.084	4.933	<.001
Expectations confirmation	Acceptance	1	0.866	N/A^a^	N/A	N/A
User satisfaction	Acceptance	0.522	0.536	0.072	7.241	<.001
Intention of use	Acceptance	0.981	0.893	0.062	15.804	<.001
Decision change	Benefit	1	0.595	N/A	N/A	N/A
Process change	Benefit	1.274	0.923	0.161	7.935	<.001
Outcome change	Benefit	1.182	0.788	0.157	7.507	<.001
Benefit	Acceptance	0.599	0.925	0.078	7.657	<.001
Acceptance	Benefit	0.599	0.388	0.078	7.657	<.001

^a^N/A: not applicable.

**Table 5:** Squared multiple correlations.

Variables	Estimate
Perceived ease of use	0.538
Benefit	0.932
Outcome change	0.621
Process change	0.851
Decision change	0.491
Acceptance	0.89
Expectations confirmation	0.75
Intention of use	0.797
User satisfaction	0.853

## Discussion

### Main Findings

User acceptance was established as central to AI-enabled clinical decision support system success in the evaluation framework. A 28-item measurement instrument was evaluated, yielding an instrument that quantifies 6 variables: *System quality*, *Information quality*, *Service quality*, *Perceived ease of use*, *User acceptance*, and *Perceived benefit*.

### User Acceptance is the Central Dimension

User acceptance is the traditional focus of evaluation in determining the success of an information system [15,17,32]. User acceptance is a synthesized concept—we used expectation confirmation, user satisfaction, and intention of use as secondary indicators. The item *system usage* was removed; DeLone and McLean [38] suggested that “intention to use,” that is, *intention of use* in our study, may be a worthwhile alternative measure in some contexts. Our work demonstrated that the use or nonuse of AI-enabled clinical decision support systems is not a universal success criterion. Therefore, the item was removed from the measurement instrument. The nature of health care settings, wherein diverse perspectives, power asymmetry, and politically led changes co-exist, supports this approach [26]. The use of an AI-enabled clinical decision support system tends to be mandatory, thus it is difficult to interpret users’ evaluations with respect to system usage. Our model demonstrated that User *Acceptance* of AI-enabled clinical decision support systems was directly determined by *Perceived ease of use*, *Information quality*, *Service quality*, and *Perceived benefit*.

### Perceived Ease of Use

In this study, perceived ease of use encompassed human–computer interaction (eg, user interface, data entry, information display, legibility, response time), ease of learning, and workflow integration [17,56,57]. *Perceived ease of use* was a mediation variable between *System quality*, *Information quality*, and *Acceptanc*e. *System quality* did not directly affect user *Acceptance*, but indirectly exerted influence through *Perceived ease of use*, principally because clinicians’ intuitive feelings of ease of use are fixed on external, tangible, and accessible features. Engineering-oriented performance characteristics of an AI-enabled clinical decision support system and necessary supporting functionalities are not their main concerns.

### Information Quality

*Information quality* refers to reliable and valid suggestions, provided by an AI-enabled clinical decision support systems, and directly and indirectly affected user *Acceptance*. Suggestions without reliability or validity not only reflects low diagnostic performance of AI-enabled clinical decision support systems but also may excessively interrupt daily work [36,58], negatively affecting ease of use and further lowering user acceptance.

### Service Quality

*Service quality* required by clinicians emphasizes knowledge updating for timeliness and system improvement [9,56].

### Perceived Benefit

*Perceived benefit* and user *Acceptance* were interrelated; and clinicians are always concerned with the usefulness of AI-enabled clinical decision support system adoption for themselves, groups, and patients [19]. AI-enabled clinical decision support system products with anticipated benefits are more likely to be accepted by clinicians. As demonstrated in our study, *Perceived benefit* was not the conclusive criterion of AI-enabled clinical decision support system success even if it could be measured with precision [59]. There will be a comparison between assumptions and expectations of personal preference with perceived benefit [36]. When clinicians are not willing to accept a new AI-enabled clinical decision support systems, the system will face adoption difficulties in clinical practice even if the system is considered to be a benefit to quality of care and patients’ outcomes in general.

### Recommendations of Benefit Measures for AI-Enabled Clinical Decision Support Systems

#### Decision Changes

We recommend using *Decision change* as an outcome measure rather than appropriate decisions. Decision change for AI-enabled clinical decision support system usage underlines decision inconsistency between system and human. These decision-making suggestions might correct users’ clinical orders, particularly for those who have insufficient practical experience [21]. Consequently, measuring user decision change (eg, tests cancel, order optimization) is more straightforward than measuring appropriate decisions.

#### Process Changes

Process change, which is similar to perceived usefulness [39], mainly covers individual, group, or organization levels of performance improvement. This study used knowledge, skills, confidence [17,25,60-62], and work efficiency [17,61] as indicators of individual performance and used quality of health care and documentation [57,62-66] as indicators of group or organization performance.

#### Outcome Changes

Outcome measures tended to be complicated indicators of AI-enabled clinical decision support system success, which often failed to be objective in clinical settings [15,58]. Beneficial patient outcomes from AI-enabled clinical decision support system implementations are the concern of all stakeholders. But there remains a paucity of high-quality evidence for outcome measures [19]. Consequently, although both subjective and objective measures of AI-enabled clinical decision support system success should compensate for the shortcomings of each other, our work showed that it is valuable to evaluate clinicians’ attitude toward perceived benefit for patients that can be obtained from specific AI-enabled clinical decision support system implementation under the health care contexts when objective measures are difficult to qualify.

### Limitations

This study is an innovative attempt and pilot examination of an evaluation framework in relation to AI-enabled clinical decision support system success. This evaluation framework is widely applicable, with a broad scope in clinically common and multidisciplinary interoperable scenarios. In order to test the validity of the variables and the hypotheses about their relationships, an empirical methodology was needed. Specifically, the items of the measurement instrument were developed targeting diagnostic AI-enabled clinical decision support systems, and AI-enabled clinical decision support systems designed to support the risk assessment of the venous thromboembolism among inpatients was the focus. Thus, one potential limitation may arise due to this narrow focus. A future expanded evaluation framework would require validation among diverse populations and encompassing AI-enabled clinical decision support systems with diverse functions.

### Implications and Conclusion

This study offers unique insight into AI-enabled clinical decision support system evaluation from a user-centric perspective, and the evaluation framework can support stakeholders to understand user acceptance of AI-enabled clinical decision support system products with various functionalities. Given the commonality and interoperability of this evaluation framework, it is widely applicable in different implementations, that is, this framework can be used to evaluate success of various AI-enabled clinical decision support systems.

From a theoretical point of view, this framework can be an evaluation approach to help in describing and understanding AI-enabled clinical decision support system success with a user acceptance–centric evaluation process. There are also practical implications in terms of how this evaluation framework is applied in clinical settings. The 28-item diagnostic AI-enabled clinical decision support system success measurement instrument, divided into 6 model variables, showed good psychometric qualities. The measurement instrument can be a useful resource for health care organizations or academic institutions designing and conducting evaluation projects on specific AI-enabled clinical decision support systems. At the same time, if the measurement instrument is to be used for AI-enabled clinical decision support system products with different functionalities in a specific scenario, item modifications, cross-cultural adaptation, and tests of reliability and validity testing (in accordance with scale development guidelines [52]) is needed.

## Appendix

Multimedia Appendix 1 Evaluation target of model variables.

Multimedia Appendix 2 Characteristics of the Delphi expert panel.

Multimedia Appendix 3 Sociodemographic characteristics of respondents.

Multimedia Appendix 4 Structure matrix of measurement instrument.

Multimedia Appendix 5 Component correlation matrix.

Multimedia Appendix 6 Standardized factor loading of the measurement instrument.

Multimedia Appendix 7 Parameter estimation of error in measurement.

Multimedia Appendix 8 Standardized total effects.

Multimedia Appendix 9 Standardized direct effects.

Multimedia Appendix 10 Standardized indirect effects.

## Abbreviations

AI: artificial intelligence
