# Supplementary material for: Evaluation Framework for Successful Artificial Intelligence–Enabled Clinical Decision Support Systems: Mixed Methods Study
Source: J Med Internet Res. 2021 Jun 2;23(6):e25929. doi: 10.2196/25929 (PMC8209524; doi:10.2196/25929)
Supplement: Multimedia Appendix 1 [file jmir_v23i6e25929_app1.docx]

Appendix 1 Evaluation target of model variables

| Model Variables | Reference and evaluation target |
| --- | --- |
| System quality | Engineering-oriented performance characteristics of the system [1], and required functionality to support the work in question |
| Information quality | The quality of AI+CDSS output from the perspective of users of the information [1]. The information quality of diagnostic AI+CDSS mainly focus on the diagnostic performance, *e.g.* reliability, validity |
| Service quality | The overall support delivered by the AI+CDSS developers [2], especially to services as knowledge updating, and system modifications. |
| Perceived ease of use | The degree to which a user believes that using a particular AI+CDSS would be free from difficulty or great effort [3]. |
| User acceptance | Is a subjective evaluation of the various consequences [4]. In this study, we constructed user acceptance as a synthesized concept, which includes *expectations confirmation* [5; 6], *user satisfaction* [7-12], and *intention of use* ^[2; 5; 6]^. *Expectations confirmation* refers to the expectation-performance discrepancy, that is users’ confirmation of expectation from prior AI+CDSS use. Expectation provides the baseline level, against which confirmation is evaluated by users to determine their use experience [13]. *User satisfaction* was conceptualized as the affective reactions of individuals toward the use of AI+CDSS in general, refers to the extent to which a system is being perceived as consistent with the users’ needs, values and experiences [14]. *Intention of use* is defined as the degree to which a user’s acceptance and subjective attitudes towards a particular AI+CDSS use. It refers to the behavioral intention rather than actual system usage [15]. |
| Perceived benefit | Is an idealized comprehensive measure of the sum of all benefits, less all costs, attributed to the use of an AI+CDSS [4]. *Clinical decision change*, *care process change*, and *patient outcome change* were assessed as three steps of benefits evaluation based on the IVCT. *Clinical decision change* is defined as individual’s decision on clinical tests or diagnosis was corrected or not. *Care process change* refers to individual’s job performance, group’s or organization’s performance ^[3; 4]^, which covers the connotation of perceived usefulness. *Patient outcome change* includes clinical outcomes and patients report outcomes, *e.g.*, morbidity, mortality, quality of life. |

Reference

1. DeLone, W. H., McLean, E. R. Information Systems Success: The Quest for the Dependent Variable. *Information Systems Research* 1992; 3(1);60-95. <http://dx.doi.org/10.1287/isre.3.1.60>.

2. DeLone, W. H., McLean, E. R. The DeLone and McLean Model of Information Systems Success: A Ten-Year Update. *Journal of Management Information Systems* 2003; 19(4);9-30. <http://dx.doi.org/10.1080/07421222.2003.11045748>.

3. Davis, F. D. Perceived Usefulness, Perceived Ease of Use, and User Acceptance of Information Technology. *MIS Quarterly* 1989; 13(3). <http://dx.doi.org/10.2307/249008>.

4. Seddon, P. B. A respecification and extension of the DeLone and McLean model of IS success. *Information Systems Research* 1997; 8(3);240-253. <http://dx.doi.org/10.2307/23010940>.

5. Kilsdonk, E.,Peute, L. W., Jaspers, M. W. M. Factors influencing implementation success of guideline-based clinical decision support systems: A systematic review and gaps analysis. *Int. J. Med. Inform.* 2017; 98;56-64. <http://dx.doi.org/10.1016/j.ijmedinf.2016.12.001>.

6. Heselmans, A.,Van de Velde, S.,Donceel, P.,Aertgeerts, B., Ramaekers, D. Effectiveness of electronic guideline-based implementation systems in ambulatory care settings - a systematic review. *Implementation Science* 2009; 4. <http://dx.doi.org/10.1186/1748-5908-4-82>.

7. Cresswell, K.,Majeed, A.,Bates, D. W., Sheikh, A. Computerised decision support systems for healthcare professionals: an interpretative review. *Inform. Prim. Care* 2012(20);115-128.

8. Bright, T. J.,Wong, A.,Dhurjati, R.,Bristow, E.,Bastian, L.,Coeytaux, R. R., et al. Effect of clinical decision-support systems: a systematic review. *Ann Intern Med* 2012; 157(1);29-43. <http://dx.doi.org/10.7326/0003-4819-157-1-201207030-00450>.

9. Souza, N. M.,Sebaldt, R. J.,Mackay, J. A.,Prorok, J. C.,Weise-Kelly, L.,Navarro, T., et al. Computerized clinical decision support systems for primary preventive care: a decision-maker-researcher partnership systematic review of effects on process of care and patient outcomes. . *Implement Sci.* 2011; 6(87). <http://dx.doi.org/10.1186/1748-5908-6-91>.

10. Nieuwlaat, R.,Connolly, S. J.,Mackay, J. A.,Weise-Kelly, L.,Navarro, T.,Wilczynski, N. L., et al. Computerized clinical decision support systems for therapeutic drug monitoring and dosing: a decision-maker-researcher partnership systematic review. *Implementation science : IS* 2011; 6;90. <http://dx.doi.org/10.1186/1748-5908-6-90>.

11. Carter, J.,Sandall, J.,Shennan, A. H., Tribe, R. M. Mobile phone apps for clinical decision support in pregnancy: a scoping review. *BMC Med. Inform. Decis. Mak.* 2019; 19(1);13. <http://dx.doi.org/10.1186/s12911-019-0954-1>.

12. Fathima, M.,Peiris, D.,Naik-Panvelkar, P.,Saini, B., Armour, C. L. Effectiveness of computerized clinical decision support systems for asthma and chronic obstructive pulmonary disease in primary care: a systematic review. *BMC Pulm. Med.* 2014(14);189. <http://dx.doi.org/10.1186/1471-2466-14-189>.

13. Bhattacherjee, A. Understanding Information Systems Continuance: An Expectation-Confirmation Model. *MIS Quarterly* 2001; 25(3);351-370. <http://dx.doi.org/10.2307/3250921>.

14. Said, S.,Al-Gahtani, Malcolm, K. Attitudes, satisfaction and usage: factors contributing to each in the acceptance of information technology. *Behav. Inf. Technol.* 1999; 18(4);277-297. <http://dx.doi.org/10.1080/014492999119020>.

15. Jackson, C. M.,Chow, S., Leitch, R. A. Toward an Understanding of the Behavioral Intention to Use an Information System *Decision Sciences* 1997; 28(2);357-389.
