# Supplementary material for: Evaluation Framework for Successful Artificial Intelligence–Enabled Clinical Decision Support Systems: Mixed Methods Study
Source: J Med Internet Res. 2021 Jun 2;23(6):e25929. doi: 10.2196/25929 (PMC8209524; doi:10.2196/25929)
Supplement: Multimedia Appendix 2 [file jmir_v23i6e25929_app2.docx]

Appendix 2 Characteristics of Expert Panel from the Delphi Process

| No. | Professional title | Degree | Working yrs | AI+CDSS practice yrs | Departments | Ca | Cs | Cr |
| --- | --- | --- | --- | --- | --- | --- | --- | --- |
| 1 | Senior | PhD | ≥10 | ≥5 | Clinical practitioner | 5.0 | 5.0 | 5.0 |
| 2 | Senior | Master | ≥25 | ≥5 | Clinical practitioner | 4.0 | 4.0 | 4.0 |
| 3 | Senior | PhD | ≥30 | ≥3 | Clinical practitioner | 4.0 | 3.0 | 3.5 |
| 4 | Senior | PhD | ≥30 | ≥5 | Clinical practitioner | 4.0 | 4.0 | 4.0 |
| 5 | Senior | Master | ≥25 | ≥10 | Hospital CIO | 5.0 | 5.0 | 5.0 |
| 6 | Senior | Master | ≥30 | ≥10 | Hospital CIO | 4.0 | 5.0 | 4.5 |
| 7 | Senior | Master | ≥20 | ≥10 | Hospital CIO | 4.0 | 5.0 | 4.5 |
| 8 | Senior | Master | ≥10 | ≥10 | Hospital CIO | 5.0 | 5.0 | 5.0 |
| 9 | Middle level | PhD | ≥10 | ≥10 | IT engineers in medical IS enterprises | 5.0 | 5.0 | 5.0 |
| 10 | Middle level | PhD | ≥10 | ≥10 | IT engineers in medical IS enterprises | 5.0 | 4.0 | 4.5 |
