# Supplementary material for: Evaluation Framework for Successful Artificial Intelligence–Enabled Clinical Decision Support Systems: Mixed Methods Study
Source: J Med Internet Res. 2021 Jun 2;23(6):e25929. doi: 10.2196/25929 (PMC8209524; doi:10.2196/25929)
Supplement: Multimedia Appendix 3 [file jmir_v23i6e25929_app3.docx]

Appendix 3 Sociodemographic characteristics of respondents

| Characteristics | n (%) | Characteristics | n (%) |
| --- | --- | --- | --- |
| Age group, years |  | Gender |  |
| *18-25* | 21 (13.46) | *Male* | 80 (51.28) |
| *26-30* | 52 (33.33) | *Female* | 76 (48.72) |
| *31-35* | 38 (24.36) | Professional post |  |
| *36-40* | 25 (16.03) | *Doctor* | 133 (85.26) |
| *41-60* | 20 (12.82) | *Nurse* | 23 (14.74) |
| Education |  | Professional title |  |
| *Bachelor* | 59 (37.82) | *Junior* | 91 (58.33) |
| *Master* | 53 (33.97) | *Intermediate* | 46 (29.49) |
| *PhD* | 44 (28.21) | *Senior* | 19 (12.18) |
