# Supplementary material for: Evaluation Framework for Successful Artificial Intelligence–Enabled Clinical Decision Support Systems: Mixed Methods Study
Source: J Med Internet Res. 2021 Jun 2;23(6):e25929. doi: 10.2196/25929 (PMC8209524; doi:10.2196/25929)
Supplement: Multimedia Appendix 4 [file jmir_v23i6e25929_app4.docx]

Appendix 4 Structure Matrix for EFA of measurement instrument

| Item | Ease of Use | System Quality | Information Quality | Service Quality | Decision Change | Process Change | Outcome Change | Acceptance | Communalities |
| --- | --- | --- | --- | --- | --- | --- | --- | --- | --- |
| 1 | **.876** | .359 | .402 | .216 | .371 | .468 | .313 | .465 | .810 |
| 2 | **.882** | .475 | .268 | .262 | .216 | .475 | .277 | .442 | .847 |
| 3 | **.719** | .656 | .279 | .225 | .405 | .530 | .234 | .659 | .717 |
| 4 | **.727** | .567 | .446 | .325 | .298 | .416 | .315 | .540 | .747 |
| 5 | **.765** | .576 | .420 | .374 | .324 | .433 | .331 | .544 | .761 |
| 6 | **.808** | .351 | .678 | .245 | .309 | .434 | .366 | .588 | .849 |
| 7 | .582 | **.815** | .363 | .190 | .343 | .363 | .102 | .589 | .751 |
| 8 | .458 | **.900** | .390 | .296 | .219 | .289 | .271 | .437 | .861 |
| 9 | .423 | .447 | **.881** | .431 | .318 | .417 | .306 | .454 | .848 |
| 10 | .662 | .292 | **.785** | .548 | .466 | .590 | .438 | .622 | .778 |
| 22 | .297 | .274 | .411 | **.931** | .401 | .477 | .446 | .466 | .890 |
| 23 | .389 | .227 | .498 | **.909** | .343 | .537 | .488 | .532 | .859 |
| 15 | .398 | .195 | .347 | .372 | **.935** | .652 | .430 | .558 | .888 |
| 16 | .317 | .282 | .293 | .362 | **.936** | .593 | .471 | .526 | .898 |
| 12 | .458 | .408 | .428 | .356 | .532 | **.813** | .355 | .632 | .766 |
| 13 | .652 | .456 | .430 | .584 | .563 | **.859** | .575 | .650 | .837 |
| 14 | .657 | .416 | .379 | .499 | .584 | **.889** | .543 | .623 | .850 |
| 17 | .471 | .128 | .370 | .417 | .591 | **.840** | .501 | .562 | .840 |
| 18 | .549 | .213 | .484 | .573 | .464 | **.760** | .656 | .443 | .820 |
| 21 | .423 | .168 | .363 | .429 | .575 | **.819** | .520 | .634 | .742 |
| 19 | .535 | .326 | .390 | .449 | .532 | .613 | **.884** | .649 | .883 |
| 20 | .411 | .240 | .378 | .540 | .564 | .693 | **.902** | .556 | .879 |
| 24 | .585 | .472 | .453 | .534 | .637 | .756 | .437 | **.899** | .867 |
| 25 | .574 | .571 | .456 | .458 | .583 | .634 | .564 | **.852** | .801 |
| 26 | .534 | .412 | .477 | .447 | .587 | .785 | .463 | **.870** | .852 |
| 27 | .471 | .346 | .431 | .616 | .418 | .565 | .584 | **.828** | .839 |
| 28 | .688 | .549 | .483 | .409 | .647 | .726 | .509 | **.891** | .859 |
| 29 | .673 | .427 | .427 | .516 | .682 | .784 | .425 | **.822** | .824 |

Note: Extraction Method: Principal Component Analysis. Rotation Method: Promax with Kaiser normalization. Major loadings for each item are bolded.
