# Supplementary material for: Evaluation Framework for Successful Artificial Intelligence–Enabled Clinical Decision Support Systems: Mixed Methods Study
Source: J Med Internet Res. 2021 Jun 2;23(6):e25929. doi: 10.2196/25929 (PMC8209524; doi:10.2196/25929)
Supplement: Multimedia Appendix 5 [file jmir_v23i6e25929_app5.docx]

Appendix 5 Component Correlation Matrix

| Component | 1 | 2 | 3 | 4 | 5 | 6 | 7 | 8 |
| --- | --- | --- | --- | --- | --- | --- | --- | --- |
| 1. Process Change | 1.000 | .583 | .670 | .342 | .533 | .652 | .560 | .425 |
| 2. Ease of Use | .583 | 1.000 | .634 | .532 | .355 | .404 | .393 | .501 |
| 3. Acceptance | .670 | .634 | 1.000 | .518 | .455 | .602 | .449 | .500 |
| 4. System Quality | .342 | .532 | .518 | 1.000 | .241 | .279 | .178 | .288 |
| 5. Service Quality | .533 | .355 | .455 | .241 | 1.000 | .359 | .525 | .441 |
| 6. Decision Change | .652 | .404 | .602 | .279 | .359 | 1.000 | .433 | .335 |
| 7. Outcome Change | .560 | .393 | .449 | .178 | .525 | .433 | 1.000 | .379 |
| 8. Information Quality | .425 | .501 | .500 | .288 | .441 | .335 | .379 | 1.000 |
