# Supplementary material for: Evaluation Framework for Successful Artificial Intelligence–Enabled Clinical Decision Support Systems: Mixed Methods Study
Source: J Med Internet Res. 2021 Jun 2;23(6):e25929. doi: 10.2196/25929 (PMC8209524; doi:10.2196/25929)
Supplement: Multimedia Appendix 6 [file jmir_v23i6e25929_app6.docx]

Appendix 6 Standardized Factor Loading of Measurement Instrument

| Variables | Item | Coef. | Std. Error | *z* | *p* | Std. Estimate |
| --- | --- | --- | --- | --- | --- | --- |
| Ease of Use | 1 | 1.000 | - | - | - | 0.807 |
|  | 2 | 0.884 | 0.080 | 11.110 | 0.000 | 0.795 |
|  | 3 | 1.061 | 0.102 | 10.399 | 0.000 | 0.757 |
|  | 4 | 0.988 | 0.100 | 9.917 | 0.000 | 0.729 |
|  | 5 | 1.017 | 0.101 | 10.024 | 0.000 | 0.736 |
|  | 6 | 0.810 | 0.076 | 10.680 | 0.000 | 0.772 |
| System Quality | 7 | 1.000 | - | - | - | 0.891 |
|  | 8 | 0.787 | 0.089 | 8.885 | 0.000 | 0.736 |
| Information Quality | 9 | 1.000 | - | - | - | 0.660 |
|  | 10 | 1.425 | 0.169 | 8.420 | 0.000 | 0.884 |
| Service Quality | 22 | 1.000 | - | - | - | 0.835 |
|  | 23 | 0.921 | 0.081 | 11.299 | 0.000 | 0.910 |
| Perceived Benefit | 12 | 1.000 | - | - | - | 0.734 |
|  | 13 | 1.144 | 0.104 | 10.965 | 0.000 | 0.855 |
|  | 14 | 1.109 | 0.101 | 10.999 | 0.000 | 0.858 |
|  | 15 | 0.933 | 0.103 | 9.039 | 0.000 | 0.716 |
|  | 16 | 0.897 | 0.106 | 8.433 | 0.000 | 0.671 |
|  | 17 | 0.871 | 0.086 | 10.070 | 0.000 | 0.791 |
|  | 18 | 0.747 | 0.079 | 9.518 | 0.000 | 0.751 |
|  | 19 | 0.869 | 0.091 | 9.562 | 0.000 | 0.754 |
|  | 20 | 0.909 | 0.092 | 9.911 | 0.000 | 0.780 |
|  | 21 | 0.890 | 0.088 | 10.064 | 0.000 | 0.791 |
| Acceptance | 24 | 1.000 | - | - | - | 0.910 |
|  | 25 | 0.900 | 0.056 | 16.196 | 0.000 | 0.859 |
|  | 26 | 0.846 | 0.050 | 16.960 | 0.000 | 0.875 |
|  | 27 | 0.773 | 0.061 | 12.688 | 0.000 | 0.763 |
|  | 28 | 1.027 | 0.055 | 18.696 | 0.000 | 0.908 |
|  | 29 | 0.926 | 0.054 | 17.038 | 0.000 | 0.877 |
