# Supplementary material for: Evaluation Framework for Successful Artificial Intelligence–Enabled Clinical Decision Support Systems: Mixed Methods Study
Source: J Med Internet Res. 2021 Jun 2;23(6):e25929. doi: 10.2196/25929 (PMC8209524; doi:10.2196/25929)
Supplement: Multimedia Appendix 7 [file jmir_v23i6e25929_app7.docx]

Appendix 7 Parameter estimation of error in measurement

| Variables | Estimate | S.E. | C.R. | *P* |
| --- | --- | --- | --- | --- |
| System Quality | 2.241 | .255 | 8.803 | <.001 |
| Information Quality | 1.104 | .125 | 8.803 | <.001 |
| Service Quality | 1.512 | .172 | 8.803 | <.001 |
| Ease of use | .446 | .051 | 8.803 | <.001 |
| Acceptance | .172 | .072 | 2.391 | .017 |
| Benefit | .045 | .022 | 1.996 | .046 |
| Expectations Confirmation | .521 | .072 | 7.191 | <.001 |
| User Satisfaction | .217 | .029 | 7.534 | <.001 |
| Intention of Use | .382 | .057 | 6.688 | <.001 |
| Decision Change | .939 | .120 | 7.796 | <.001 |
| Process Change | .185 | .042 | 4.377 | <.001 |
| Outcome Change | .556 | .074 | 7.509 | <.001 |
