# Supplementary material for: Evaluation Framework for Successful Artificial Intelligence–Enabled Clinical Decision Support Systems: Mixed Methods Study
Source: J Med Internet Res. 2021 Jun 2;23(6):e25929. doi: 10.2196/25929 (PMC8209524; doi:10.2196/25929)
Supplement: Multimedia Appendix 9 [file jmir_v23i6e25929_app9.docx]

Appendix 9 Standardized Direct Effects

|  | Service Quality | Information Quality | System Quality | Ease of use | Benefit | Acceptance |
| --- | --- | --- | --- | --- | --- | --- |
| Ease of use | .000 | .405 | .446 | .000 | .000 | .000 |
| Benefit | .000 | .000 | .000 | .000 | .000 | .925 |
| Outcome Change | .000 | .000 | .000 | .000 | .788 | .000 |
| Process Change | .000 | .000 | .000 | .000 | .923 | .000 |
| Decision Change | .000 | .000 | .000 | .000 | .595 | .000 |
| Acceptance | .232 | .099 | .000 | .325 | .388 | .000 |
| Expectations Confirmation | .000 | .000 | .000 | .000 | .000 | .866 |
| Intention of Use | .000 | .000 | .000 | .000 | .000 | .893 |
| User Satisfaction | .000 | .000 | .000 | .000 | .000 | .536 |
