# Supplementary material for: Evaluation Framework for Successful Artificial Intelligence–Enabled Clinical Decision Support Systems: Mixed Methods Study
Source: J Med Internet Res. 2021 Jun 2;23(6):e25929. doi: 10.2196/25929 (PMC8209524; doi:10.2196/25929)
Supplement: Multimedia Appendix 10 [file jmir_v23i6e25929_app10.docx]

Appendix 10 Standardized Indirect Effects

|  | Service Quality | Information Quality | System Quality | Ease of use | Benefit | Acceptance |
| --- | --- | --- | --- | --- | --- | --- |
| Ease of use | .000 | .000 | .000 | .000 | .000 | .000 |
| Benefit | .334 | .332 | .209 | .469 | .559 | .517 |
| Outcome Change | .263 | .262 | .165 | .370 | .441 | 1.136 |
| Process Change | .308 | .306 | .193 | .432 | .516 | 1.330 |
| Decision Change | .199 | .198 | .124 | .279 | .333 | .858 |
| Acceptance | .130 | .260 | .226 | .182 | .217 | .559 |
| Expectations Confirmation | .313 | .311 | .196 | .439 | .523 | .484 |
| Intention of Use | .322 | .321 | .202 | .453 | .540 | .499 |
| User Satisfaction | .325 | .323 | .203 | .456 | .544 | .866 |
